# TRIB3-P62 interaction, diabetes and autophagy

**DOI:** 10.18632/oncotarget.6108

**Published:** 2015-10-13

**Authors:** Fang Hua, Zhuo-Wei Hu

**Affiliations:** Immunology and Cancer Pharmacology Group, State Key Laboratory of Bioactive Substance and Function of Natural Medicines, Institute of Materia Medica; Chinese Academy of Medical Sciences and Peking Union Medical College, Beijing, P.R. China

**Keywords:** diabetes, tumorigenesis, metastasis, protein-protein interactions, α-helical peptide

Type 2 diabetes (T2D) and cancer are two common diseases with tremendous impact on health worldwide. Epidemiologic evidence suggests that cancer incidence is associated with diabetes as well as certain diabetic risk factors and diabetic medications. Generally, hyperinsulinemia and high level of circulating Insulin-like growth factor 1 (IGF-1) contribute to cancer development through the insulin/IGF's growth-promoting effect [1, 2]. Medications used to treat T2D can influence some of these factors and may decrease or increase the risk of cancer and cancer-related mortality [3]. Although insulin and IGF-1 have been long assumed as biological links between energy metabolic disorders and cancers, the underlining mechanism remains incompletely understood.

Tribbles 3 (TRIB3) is a member of Tribbles homolog family which belongs to the pseudokinase. TRIB3 contains a variant kinase domain, but lacks the adenosine 5′-triphosphate (ATP)-binding and catalytic core motifs. Several studies suggest that TRIB3 expression is increased in response to various stresses, including nutrient deficiency, endoplasmic reticulum stress, oxidative stress and many metabolic stresses, such as hypoxia, hyperglycemia and hyperinsulinemia. Emerging literatures have described important effects for TRIB3 in the regulation of glucose and lipid homeostasis. Recently, several groups including us identify TRIB3 being a crucial player in the regulation of tumorigenesis and tumor progression. We thus suspect TRIB3 acts as a bridge to connect the metabolic risk factors to tumor promotion.

Indeed, we demonstrate that metabolic stresses enhance the TRIB3-mediated malignancy-promoting actions [4]. TRIB3 directly interacts with autophagic receptor P62 to interfere with the binding of P62 to LC3 and to ubiquitinated proteins, which results in the defect of P62-mediated selective autophagy and autophagy-dependent clearance of ubiquitinated proteins, and subsequently bothers the ubiquitin-proteasome-dependent degradation. A number of cancer-promoting factors including EGFR, COX-2, MMP1/2, MT-MMP, c-Myc, as well as Snail and Twist accumulate in the cells due to the dysfunction of the two major routes for intracellular protein degradation. Interrupting the TRIB3/P62 interaction by a P62-derived α-helical peptide inhibits tumor initiation, growth and metastasis, and thus increases animal survival through restoring the selective autophagy degradation and accelerating the ubiquitin proteasome system (UPS) clearance of the tumor-promoting factors.

Much of the research state that insulin and IGF-1 are the critical biologic link between diabetes and cancer depending on their pro-mitogenic, pro-angiogenesis, pro-migration and pro-glycolysis effects. However, there is controversy regarding to the pro-mitogenic effects as the mechanisms of insulin/IGF-1 for cancer development. For instance, Weinberg points out that the primary role of insulin/IGF-1 is not to turn on the Warburg effect or promote proliferation but suppress cell-suicide mechanisms [1]. In fact, recent clinical trials show that targeting insulin/IGF-1 signal does not produce satisfactory efficacy against cancers [2]. Our studies document that antagonism of insulin/IGF signaling producing an unsatisfactory efficacy against cancer may be attributed to the high expression level of TRIB3 induced by a diversity of metabolic stresses surrounding cancer cells and tumor tissue. In our opinion, TRIB3 seems to act as another molecular link connecting several common threads (stress factors) to cancer development.

**Figure 1:**
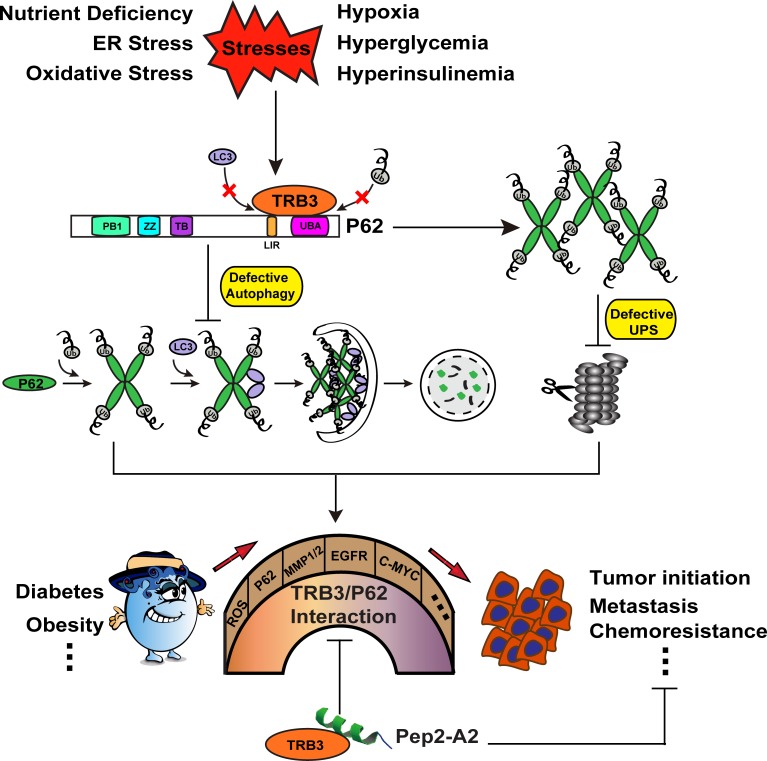
Schematic diagram of the TRIB3-P62 interaction in the connection of diabetes to cancer promotion Many stresses can increase the expression of TRIB3 and enhance the interaction between TRIB3 and P62. The TRIB3-P62 interaction abrogates the binding of LC3 and ubiquitinated substrates to P62, which induces the blockage of autophagic flux and subsequent UPS defect. UPS and autophagy are considered as the last line of defense in protein quality control. Dysfunction of the two degradation systems results in the accumulation of ROS and a multitude of cancer-promoting factors, which act as bricks or stones to build a bridge for connecting diabetes to tumor promotion. P62-derived α-helical peptide (Pep2-A2) can interfere with the TRIB3-P62 interaction and produce a potent antitumor effect, suggesting that the TRIB3-P62 interaction is a candidate therapeutic target against cancer.

The role of autophagy in cancer is quite complicated and still somewhat controversial. Some of the view considered that autophagy appears to be tumor suppressive during cancer development, but contributes to tumor cell survival during cancer progression. In our recent study and previous works [4, 5], we find although the autophagic signaling molecules are activated in some pathologic situations, a huge amount of insoluble P62 accumulates in cells and tissues, which indicates a failure of autophagic flux and accounts for the initiation and progression of cancer. Our observations highlights that unrestricted and effective autophagic flux, together with moderately activated autophagic signaling are essential for tumor inhibition. Previous studies had emphasized the critical roles of P62 in cancers. In our recent study, the harmful effects of TRIB3 are mainly caused by P62. One should ask that can P62 be chosen as a more direct cancer therapy target. However, it's a challenge to selectively eliminate P62 from cancer cells with no (or much less) effect on its autophagic cargo functions in normal cells. Indeed, silencing P62 even causes detrimental effects in response to IGF because the depletion of P62 from cells damages its physiological functions and interferes with the selective autophagy clearance. In contrast, TRIB3 is a stress-induced responsive protein that expresses highly in human cancer or stressed cells, but does not express in normal cells. Our study confirms that the TRIB3/P62 interaction is the critical node for their tumor promotion effects, which provides a proof-of-concept for targeting this interaction as a therapeutic strategy against cancers.

The TRIB3-P62-autophagy and UPS pathways identified in our study may be critical not only for insulin/ IGF-fueled tumor development but also for stress-relevant malignant diseases. Thus, our studies have several folds of implications in further researches. First, antineoplastic agents often induce stresses in biological systems. The efficacy of chemotherapeutics may be resisted if they enhance TRIB3 expression. Namely, chemoresistance may be mediated by the agent-enhanced TRIB3/p62 interaction. Also, agents restoring autophagy by interrupting the TRIB3/P62 interaction may have additional therapeutic applications, particularly for the treatment of diabetic or overnutrition-induced chronic diseases as well as synergistic usage for circumventing chemoresistance [6, 7]. Third, given that TRIB3 expression is enhanced by a variety of stresses and the TRIB3/p62 interaction may occur in tumor or stressed cells but not or less in normal cells, it is likely that drugs targeting this interaction have minimal toxicity.
